# A Hybrid Approach to Tea Crop Yield Prediction Using Simulation Models and Machine Learning

**DOI:** 10.3390/plants11151925

**Published:** 2022-07-25

**Authors:** Dania Batool, Muhammad Shahbaz, Hafiz Shahzad Asif, Kamran Shaukat, Talha Mahboob Alam, Ibrahim A. Hameed, Zeeshan Ramzan, Abdul Waheed, Hanan Aljuaid, Suhuai Luo

**Affiliations:** 1 Department of Computer Engineering, University of Engineering and Technology, Lahore 58590, Pakistan; daniaalvi34@gmail.com (D.B.); m.shahbaz@uet.edu.pk (M.S.); 2 Department of Computer Science, New Campus, University of Engineering and Technology, Lahore 58590, Pakistan; shehzad@uet.edu.pk; 3 School of Information and Physical Sciences, The University of Newcastle, Newcastle 2308, Australia; suhuai.luo@newcastle.edu.au; 4 Department of Data Science, University of the Punjab, Lahore 54890, Pakistan; 5 Department of Computer Science and Information Technology, Virtual University of Pakistan, Lahore 58590, Pakistan; 6 Department of ICT and Natural Sciences, Norwegian University of Science and Technology, 7034 Trondheim, Norway; 7 National Tea and High-Value Crops Research Institute, Shinkiari, Mansehra 21300, Pakistan; abdulw900@gmail.com; 8 Computer Sciences Department, College of Computer and Information Sciences, Princess Nourah bint Abdulrahman University (PNU), P.O. Box 84428, Riyadh 11671, Saudi Arabia; haaljuaid@pnu.edu.sa

**Keywords:** crop simulation models, machine learning, AquaCrop, tea yield, crop yield prediction

## Abstract

Tea (*Camellia sinensis* L.) is one of the most highly consumed beverages globally after water. Several countries import large quantities of tea from other countries to meet domestic needs. Therefore, accurate and timely prediction of tea yield is critical. The previous studies used statistical, deep learning, and machine learning techniques for tea yield prediction, but crop simulation models have not yet been used. However, the calibration of a simulation model for tea yield prediction and the comparison of these approaches is needed regarding the different data types. This research study aims to provide a comparative study of the methods for tea yield prediction using the Food and Agriculture Organization (FAO) of the United Nations AquaCrop simulation model and machine learning techniques. We employed weather, soil, crop, and agro-management data from 2016 to 2019 acquired from tea fields of the National Tea and High-Value Crop Research Institute (NTHRI), Pakistan, to calibrate the AquaCrop simulation model and to train regression algorithms. We achieved a mean absolute error (*MAE*) of 0.45 t/ha, a mean squared error (*MSE*) of 0.23 t/ha, and a root mean square error (*RMSE*) of 0.48 t/ha in the calibration of the AquaCrop model and, out of the ten regression models, we achieved the lowest *MAE* of 0.093 t/ha, *MSE* of 0.015 t/ha, and *RMSE* of 0.120 t/ha using 10-fold cross-validation and *MAE* of 0.123 t/ha, *MSE* of 0.024 t/ha, and *RMSE* of 0.154 t/ha using the XGBoost regressor with train test split. We concluded that the machine learning regression algorithm performed better in yield prediction using fewer data than the simulation model. This study provides a technique to improve tea yield prediction by combining different data sources using a crop simulation model and machine learning algorithms.

## 1. Introduction

Agriculture or farming are the main sources of national income for many developing countries. More than 50% of the population earns their livelihood from agriculture [1,2]. An increase in the production of agricultural products is required to ensure food security and sustainable development by increasing exports of these products. Pakistan is also an agricultural country.

Pakistan is suitable for agriculture as it has all types of land and seasons. It has a 96.9% land area and 3.1% water bodies. Of the land, 80% is irrigated, and 20% is rain-nourished [2]. Moreover, 48% of the labour force is directly associated with the agricultural sector [3]. It has greatly contributed to Pakistan’s Gross Domestic Product by increasing exports [4]. The economy of any country depends on balancing its imports and exports [5]. Therefore, there is a need to improve the agricultural sector of Pakistan as it imports many agricultural products, including tea, from other countries. Tea is used on a large scale as a worldwide beverage. In the last ten years, world tea yield increased by 4.4 percent annually and reached 5.73 million tons in 2016. China’s tea production increased from 1.17 million tons to 2.44 million tons from 2007 to 2016, respectively, which increased the global output of tea [6]. At the global level, black tea production increased by 3.0 percent annually and green tea production by 5.4 percent in the last decade [6,7].

According to the FAO, the global production of black tea is estimated to rise by 2.2 percent annually over the next ten years and will reach 4.4 million tons in 2027. The global yield of green tea is projected to increase by 7.5 percent compared to black tea and reach 3.6 million tons in 2027. Due to the perceived health benefits of tea, world tea consumption increased by 4.5% to 5.5 million tons annually over the decade. In 2016, China was the largest consumer of tea, comprising 38.6 percent, and India the second-largest, accounting for 19 percent of world tea consumption [6].

Black tea consumption is estimated to increase at an annual rate of 2.5 percent to reach 4.17 million tons in 2027. This reflects the increase in consumption in tea-producing countries [3]. Pakistan is the 2nd largest country importer of tea in the world. It imports tea from 21 different countries [8]. The increase in the amount of tea imported resulted in a huge import bill, which became a big issue for a growing country like Pakistan. Therefore, to overcome this problem and increase tea production, the National Tea Research Institute under the Pakistan Agricultural Research Council (PARC) was established in 1986 on 50 acres of land in Mansehra (Khyber Pakhtunkhwa). A tea garden was established on 30 acres of land with a complete infrastructure for tea plantations. An equipped laboratory and a tea-processing unit were installed to process tea leaves [9].

**Figure 1:**
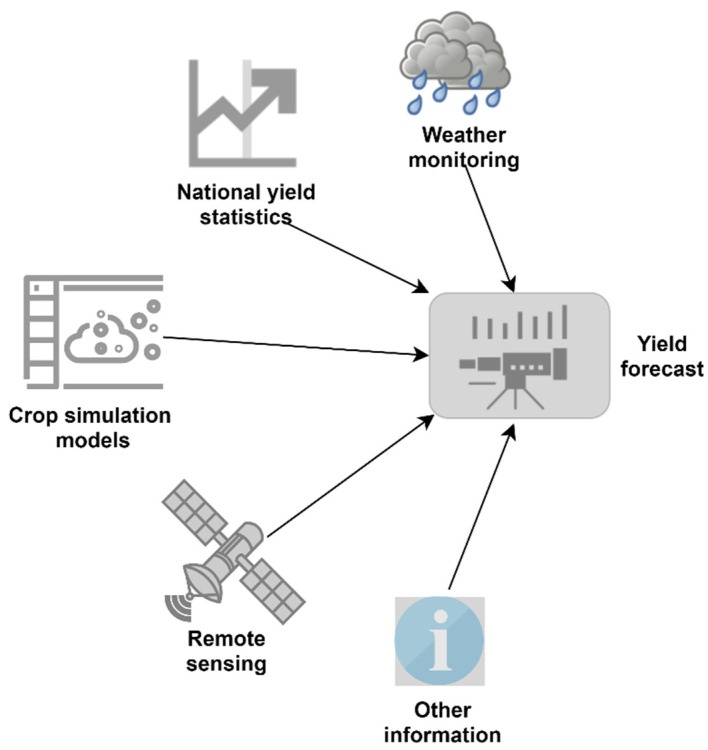
Crop yield estimation methods.

In this era of technology, many methods are being used for yield estimation of different crops [10], as shown in Figure 1. Remote sensing [11,12], crop simulation models (CSM) [13,14], and other statistical methods [15,16] are the most popular among them. These techniques are also used to find the climate impact on the yield of different types of crops [17,18].

Machine learning (ML) techniques and CSMs have been used to predict yields of several annual crops such as wheat, soybean, corn, maize, sugarcane, etc. Still, no significant work is conducted for perennial crops that carry above-ground biomass from one year to another, such as trees using CSMs [19,20]. Data mining (DM) techniques and CSMs are used for sugarcane yield prediction [13]. CSMs have been calibrated for different annual crops. Tea crop is a perennial crop; interpretation of CSM has not been made for this type of crop. Recently, a process-based model was used for tea yield prediction [18]. Various studies investigate the physiological processes of tea [21,22,23] and attempt to find the impact of climate change on tea yield [24,25,26,27,28].

In this study, we have used weather, crop, soil, and agro-management data on the growing season from 2016 to 2019 to predict tea yield in Pakistan using ML techniques and the AquaCrop simulation model. AquaCrop requires a relatively smaller number of parameters, and recently its parameterization was performed for table grapes for the growing season from 2005 to 2006 [20]. We have validated the AquaCrop simulation model by comparing its results with ML algorithms. Our study combines different types of data to improve the tea yield prediction. Using multiple data sources such as weather, crop, soil, and agro-management can improve yield prediction as only one data source cannot fully capture complete information on plant growth. This will help improve tea crop production by providing a yield forecast before plucking tea leaves. This study also provides a paradigm for CSMs for tea yield prediction in other regions. This research study aims to address the following research questions.

(1) How to calibrate the selected CSM?
(2) How do ML regression algorithms perform for tea yield prediction?
(3) Which technique from both ML and CSM performs better for tea yield prediction?

This paper is organized into the following sections: section contains relevant studies on yield prediction of different crops; Section 3 describes the data used in this study and the proposed methodology for tea yield prediction; Section 4 contains the results and discussions of our study, and Section 5 provides the conclusion of this research study.

## 2. Review of Existing Methods

Crop yield estimation is vital in providing food security to the increasing global population. It helps to improve management practices necessary to increase crop production. In the past decade, crop yield estimation has been performed using traditional statistical regression methods, ML methods, and crop models [29,30,31,32]. ML techniques have been implemented using environmental and genotype data for crop yield prediction [33]. Many crop growth models, called crop yield models or CSMs, have been developed. These terms define them as just a representation of real-world experiments [34]. These models help farmers, policymakers, and the government to maximize sustainability by providing reliable information about crop production, which is necessary for decision-making [35].

Crop growth models use plant growth processes and run these processes at multiple scales. These models help find yield during plant growth, resulting from several variables such as climate, plant density, crop management, stress factors, etc. [35,36,37,38]. Guerra et al. [39] evaluate using CSMs combined with interpolation to estimate the monthly distribution of irrigation water for cotton in Georgia. The Decision Support System for Agrotechnology Transfer model has been used to simulate the phenology process for soybean crops [40]. The Crop Environment Resource Synthesis for Wheat simulation model was calibrated for wheat yield prediction [41]. There are very few models that are being calibrated for perennial crops. The AquaCrop model has been calibrated to predict water requirements of traditional African vegetables, Amaranthus, leafy vegetables, and taro, a wetland perennial tuber crop [42,43]. The AquaCrop model was calibrated and validated for the perennial coffee crop [44].

The performance of the simulation model has been compared with DM techniques in the past. Hammer et al. [13] used the Agroecological Zone simulation model and DM techniques (random forest (RF), support vector machines (SVM), and gradient boosting machine (GBM)) to predict sugarcane yield. They concluded that DM techniques are better than the Agroecological Zone model. Contrarily, there is a simple alternative to crop models in statistical methods that uses probability for crop yield prediction. Still, these methods are localized and cannot be extended to other areas [30]. After statistical methods, ML techniques use weights instead of the likelihood for yield estimation [45]. Therefore, ML techniques are very useful when we have noisy data. They have been widely used for crop estimation and classification [17,46]. Khaki and Wang [33] used the ML approach to accurately predict corn yield and yield difference between corn hybrids and genotype or environment data and achieved an *RMSE* of 11%. ML techniques have also been used to predict a frost hazard for Zhejiang tea trees [47]. Meteorology and topography with latitude and longitude were used to estimate the damaged area. The SVM and artificial neural network (ANN) were employed for estimation. They produced 83.8% and 75% accuracy for frost damage prediction, respectively. A spatiotemporal hybrid model was developed by [48] using satellite-derived hydro-meteorological variables from 20 stations across Bangladesh between 1981 and 2020. This study used support vector regression (S) and dragonfly optimization algorithms. This hybrid model improved tea yield forecasting with least relative error value of 11%.

After ML, deep learning (DL) is an enhanced approach used in the yield estimation of many crops. You et al. [11] employed DL techniques to predict soybean yield based on remotely sensed data taken before harvest. Previous research has also used deep learning to predict corn yield [12,49]. The ANN model was also developed to predict Iran’s environmental effects and yield of black, green, and oolong tea [50]. A hybrid model combining convolutional neural network (CNN) and recurrent neural network (RNN) was used for corn and soybean yield prediction, and they concluded that these had outperformed RF, LASSO, and deep fully-connected neural networks [51]. ANN and regression were used to predict corn and soybean yield, concluding that ANN performed better [31]. A decision support system was developed using environmental and soil data to identify the best environmental conditions to increase tea production [52]. However, there is a lack of DL studies for tea yield prediction [53]. ML and DL techniques use climate, soil, crop, and satellite data to find yield patterns and estimate yield [15]. Remote sensing data allows crop status monitoring using various spectra and microwave wavelengths [54]. Climatic and satellite data have been used to predict wheat yield [55,56]. A prediction model for sorghum biomass prediction was proposed using high resolution remote temporal images as input to the SVM and multi-layer perceptron (MLP) model and the APSIM as the sorghum crop model. They concluded that the MLP model provided the most accurate result [57]. 

Many studies have been conducted to improve its products and to identify the effects on the environment, soil, climate, etc. The effect of climate change on tea yield has been analyzed, and it is observed that tea was 50% higher in the monsoon period than in spring, but the quality of tea was 50% lower during the monsoon [24]. The impact of climate change on tea production is also studied [22,25,26]. A negative impact of increased temperature and rainfall on tea yield was observed [26]. Statistical models are also used in Kenya to predict the climate effect on tea yield. These models were trained using historical temperature and precipitation data. Correlation analysis shows that tea yield and climatic variables are correlated, and rainfall and temperature are inversely related. This model predicted 70% of forecasts correctly [28].

The stochastic frontier analysis was applied to analyze irrigation water use efficiency in the tea farms of Vietnam. They found that they can save up to 57.81% of irrigation water by improving water usage [27]. Recently, a study was conducted to predict future projections of tea crops using climatic data. They observed that yields are expected to increase in China, Vietnam, and India and decrease in Kenya, Sri Lanka, and Indonesia [18].

To estimate tea yield, tea growth was simulated using data from tea cultivar TRI 2025 in Sri Lanka. They developed and calibrated a simulation model using climate, soil, and crop data collected from different climatic areas. This model simulated shoot growth, leaf area of the shoot, shoot replacement cycle, dry matter partitioning, and yield [58]. A summary of some of the above-cited work is shown in Table 1.

## 3. Methodology

### 3.1. Study Area

**Figure 2:**
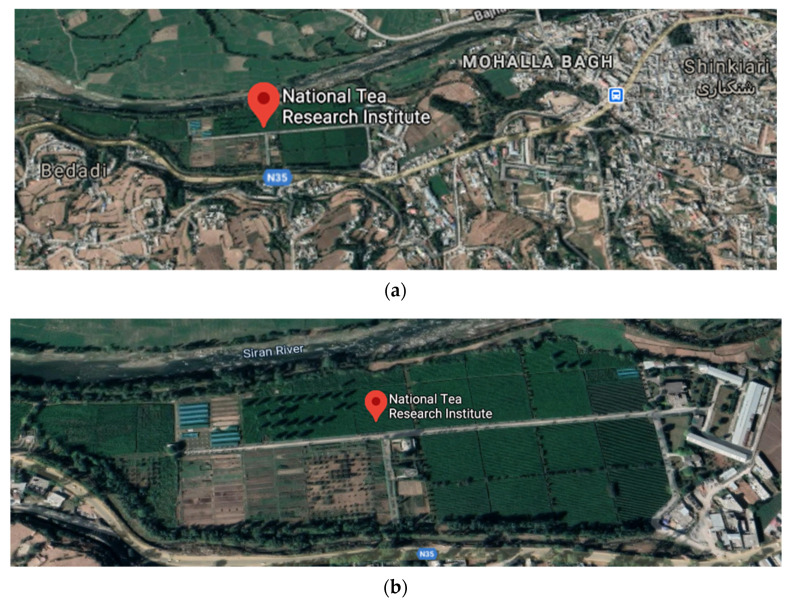
(**a**) Satellite view of Shinkiari, (**b**) satellite view of NTHRI.

We have conducted this study in Pakistan, where tea is grown. These tea fields are located at an altitude of 1000 m from the sea level and located at 34°28′0″ N, 73°16′60″ E in Pakhal valley Shinkiari, Mansehra, Pakistan, as shown in Figure 2a,b. The total area of the fields amounts to approximately 30 acres. This tea garden has an equipped soil laboratory and a black and green tea processing unit.

#### 3.1.1. Data

Weather, soil, crop, agro-management, and plucking data were acquired from the fields of NTHRI. We have also interviewed staff and local farmers to understand the tea-growing process. Tea yield data (kgs per acre) were collected during growing seasons from 2009 to 2019. The plucking data from 2016 to 2019 was more accurate and without errors. The previous year’s data was not recorded properly and lacked detailed plucking information, so we had to discard that data. The following information was collected from NTHRI tea fields regarding soil characteristics and crop management at the block level: soil type, application of fertilizers (nitrogen, phosphorus, potassium), irrigation schedule, sowing method, planting density, and crop calendar (sowing date, days to emergence, initial canopy cover, days to maturity, days to max canopy cover, and days to harvesting). 

The collected meteorological data were minimum temperature, maximum temperature, humidity, rainfall, and wind speed obtained from weather stations in the NTHRI and neighbouring regions. We have estimated evapotranspiration using the FAO Penman-Monteith equation, which uses weather parameters. These meteorological data were utilized to estimate tea yield using the AquaCrop simulation model and ML techniques.

#### 3.1.2. Pre-Processing

We collected data in a raw format that needed to be pre-processed. We cleaned this dataset and converted it to the plot level. Every sample shows yield value from one plot on a specific day, each plot of one acre. The total number of samples was 239. Meteorological data were mapped to these samples according to the given dates. These data were prepared to be fed to ML algorithms. We prepared four datasets for the CSM, including weather, crop, soil, and agro-management data. We used these dataset files to calibrate our AquaCrop model. The samples of all the types of data are given below.

##### Weather Data

Average values of weather data for min temperature, max temperature, humidity, rainfall, and solar radiation are shown in Table 2.

##### Soil Data

Soil data contains soil type, soil depth, and the water content in the soil, as shown in Table 3. We used conservative values of other parameters such as field capacity, soil saturation, wilting point, runoff, etc.

##### Crop Data

Crop data contains information about the crop calendar. It has all the information related to the sowing of the plant, plant density, growth cycle, plant age, irrigation, fertility stress, water stress, etc., as represented in Table 4.

##### Agro-Management Data

It includes data related to agro-management practices. At NTHRI, no additional management practices such as mulches, etc., are applied. We received information about irrigation water applied by conducting interviews with scientists and other staff. There is no specific irrigation schedule. The irrigation amounts and days are decided according to the requirement of a particular day.

These were the input parameters whose values were recorded. We have also used estimated values of input parameters such as minimum effective rooting depth, crop coefficients, bulk density, soil depth, field capacity, initial salinity, etc. Some variables are calibrated, including base and cut-off temperature, initial and maximum canopy cover, canopy growth coefficient (CGC), and crop calendar, including time from sowing to emergence, senescence, maturity, and harvesting.

### 3.2. Methods Implementation

**Figure 3:**
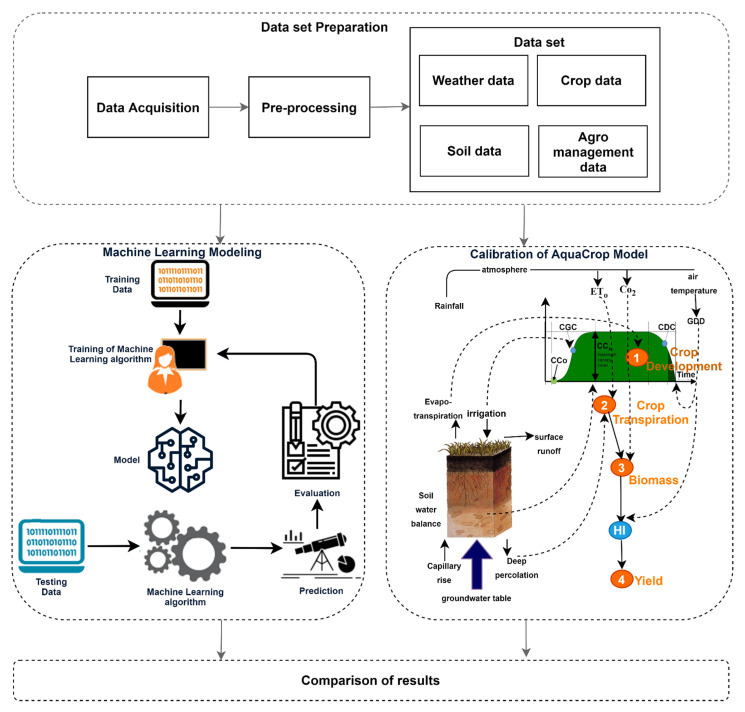
Methodology for the comparative study of tea yield prediction.

We have proposed a framework based on CSM and ML techniques to predict tea yield using weather, crop, agro-management, and soil data. Our proposed methodology has two stages. These stages work concurrently, as shown in Figure 3.

#### 3.2.1. Estimation of Tea Yield Based on the FAO AquaCrop Model 

In the first stage, for the estimation of tea yield throughout the crop cycles, the FAO AquaCrop model [59] was calibrated. This CSM has a mathematical approach for estimating yield. This approach considers crop development, crop transpiration, biomass production, and yield formation for yield estimation, as shown in Figure 3. It also considers the impact of water deficiency on yield. The AquaCrop approach is different from ML algorithms which only use statistical functions.

For yield estimation using AquaCrop, the following meteorological parameters were used as climatic input data: minimum temperature, maximum temperature, rainfall, humidity, solar radiation, and wind speed. These data were provided as daily data. AquaCrop used these meteorological parameters to estimate the reference Evapotranspiration (ET_o)_ using the Penman-Monteith method. The crop and soil characteristics defined above were also used as input for calibration of the model. An irrigation schedule was provided to overcome the rain deficiency and keep water content at field capacity.

Canopy cover (*CC*) was calculated using all these input parameters directly affected by the total amount of available water in the soil. It was calculated using the given Equation (1).

(1) $$CC=\frac{soil\;surface\;covered\;by\;the\;green\;canopy}{unit\;ground\;surface\;area}$$

This *CC* represents the land area, which is covered with green leaves. We have used an initial canopy cover of 20% and a maximum canopy cover of 95%. There was no water or fertility stress during the crop life cycle. When the *CC* increases, it affects the amount of water transpired. Transpiration rate (*Tr*) has a vital role in plant growth [60] which depends on the type of crop, energy supply, vapour pressure, and wind [61].

The product of the *Kc_Tr_* calculated crop transpiration with *ETo* and by considering a cold stress (*ks_Tr,x_*) and water stress (*Ks*) coefficient as given in Equation (2). Their value is 1 when stress does not trigger stomatal closure.

(2) $$Tr\;=\;Ks\;(Ks_{Tr,x}\;Kc_{Tr})\;ETo$$

*Kc_Tr_* is proportional to the *CC* and hence varies throughout the whole growth cycle of the plant with simulated *CC*. Water stress affects canopy development, stomal closure, and hence crop transpiration which was calculated using Equation (3) [62].

(3) $$Kc_{Tr}=Kc_{Tr,x}CC*$$

As the crop transpiration increases and the plant grows, it produces biomass which we call above-ground biomass. It is mostly given in tons/hectares and is directly proportional to crop transpiration (Σ*Tr*). The factor of proportionality is biomass water productivity (*WP*). It is normalized for climatic conditions in AquaCrop, making this *WP* valid for diverse locations, environments, and CO_2_ concentrations. The potential biomass value was calculated using Equation (4).

(4) $$B=WP*\sum^{\;}\left(\frac{Tr_{i}}{ETo_{i}}\right)$$

After this, the biomass converts to yield. The biomass is not converted into the yield; rather, a fraction is converted into yield (*Y*). A scaling factor, harvest index (*HI*), was used to calculate this yield part, representing the part of the harvested product as a percentage of the biomass. The yield was calculated using Equation (5).

(5) Y=HI ∗ B

As the water and temperature stresses vary throughout the growth cycle, the *HI* might be altered from its reference value (*HIo*) which was used in the range of 14% to 19% for tea yield. A multiplier is used with (*HIo*) to continuously adjust the effect of stresses on the *HI* as given by Equation (6).

(6) HI=multiplier HIo

For the validation of AquaCrop results, the simulated yield (t/ha) was compared to the observed (t/ha). For comparison *MAE*, *MSE*, and *RMSE* were used as performance measures.

#### 3.2.2. Estimation of Tea Yield Using ML Techniques 

In the second stage, we employed linear regression-based ML techniques. Linear regression analysis is a predictive modelling approach that finds a relationship between the independent (predictor) and the dependent (target) variable. We can represent the simplest regression equation using one dependent and independent variable by Equation (7).

(7) y=c+b∗x

where *y* is the dependent variable, which is to be estimated, *x* is the independent variable, *c* is a constant, and *b* is the regression coefficient [63]. It can be used to find the strength of the impact of independent variables on the dependent variable. It can also be used to forecast how a dependent variable changes with the change of any independent variable.

In this study, we have modelled to predict tea yield including regression algorithms, such as Linear Support Vector Regression (SVR), AdaBoost Regressor, Automatic Relevance Determination (ARD) Regression, Decision Tree Regressor, MLP Regressor, Multiple Linear Regression (MLR), Random Sample Consensus (RANSAC) Regressor, Simple Linear Regression (SLR), XGBoost, and SVM Regressor. A brief description of these regression algorithms is given below.

(a) Linear SVR

Linear SVR tries to fit the error in a specific threshold given by Equation (8). It utilizes the same rules as the SVM classifier with just a few variations. Regression produces a real number with unlimited possibilities, which is very hard to estimate. A tolerance margin in line with the SVM is set to fix this problem. The primary principle is to minimize the mistake by tolerating the mistake and individualizing the hyperplane that maximizes the margin [64].

(8) $$y=\overset{N}{\underset{i=1}{\sum }}(\alpha_{i}-\alpha_{i}{}^{*}).\langle x_{i},x\rangle +b$$

Different parameter values are used as: kernel = ‘rbf’, degree = 3, gamma = ‘scale’, coef0 = 0.0, tol = 0.001, C = 1.0, epsilon = 0.1, shrin king = True, cache_size = 200, verbose = False, max_iter = −1.

(b) AdaBoost Regressor

AdaBoost, short for Adaptive Boosting, can be used to optimize their performance in conjunction with other forms of learning algorithms. Many algorithms are slow learners and use boosted algorithms, and their output is incorporated into a weighted sum as given by Equation (9). For outliers and noisy data, AdaBoost is vulnerable [65].

(9) $$F_{T}\left(x\right)=\overset{T}{\underset{t=1}{\sum }}f_{t}\left(x\right)$$

where *f_t_* is a poor learner who takes and categorizes *x* input, the Decision Tree Regressor is used as the base estimator, and default values for all other parameters are used in the implementation.

(c) Automatic Relevance Determination (ARD) Regression

The ARD framework is an effective tool for pruning many unrelated features leading to a large explanatory subset [66].

This model estimates a value by iterations, increasing the marginal log-likelihood of the results, known as proof, in each iteration as given by Equation (10). In ARD, the basic idea is to send the function weights to independent Gaussian priors.

(10) $$p(w|\alpha )=\underset{i}{\prod }N\left(w|0,\alpha_{i}\right)$$

where *α* = (*α_i_*) is a hyperparameter vector that controls each weight value; default values of all the parameters are used in the implementation.

(d) Decision Tree Regressor

Decision trees are developed as predictive models, using a collection of binary rules to determine a target value. Each tree has branches, nodes, and leaves. It arrives at an estimate by passing data through a series of questions. At each step, each question narrows possible values and makes the model more confident in making every prediction. The model determines the order and content of questions. It can have an overfitting problem eliminated using the RF algorithm. It is implemented with a max depth of 20 with default values of all other parameters.

(e) Multilayer Perceptron (MLP) Regressor

A neural network is the simplified version of the human brain, consisting of the input, hidden, and output layers. The most used neural network model is the MLP. Different weights on each layer of a neural network allow the neural network to understand. The neurons are organized in several layers. The nodes are neurons in each layer that only accept inputs from the nodes/neurons in the previous layer and transfer the output to the next layer of nodes/neurons.

(f) Multiple Linear Regression (MLR)

It has one dependent variable and two or more independent variables. Multivariate linear regression analysis accounts for the variation in the dependent variable due to the change in independent variables synchronically given by Equation (11).

(11) $$y=\beta_{0}+\beta_{1}x_{1}+\ldots +\beta_{n}x_{n}+\varepsilon$$

The dependent variable is *y*, *x_n_* is the independent variable, the parameter is *β_n_*, and the error is *ε*. It implies the data has a normal distribution, severe value independence, linearity, and no multiple relations between independent variables [67]. We have used yield as the dependent variable and all other parameters as independent variables. The default parameters for linear regression are used in the implementation.

(g) Random Sample Consensus Regressor

The RANSAC iterates in two steps:

- Generating a hypothesis from a random sample of data (hypothesis generation)
- Verifying it with other data (hypothesis evaluation)

It is widely used in the image processing field for cleaning noisy datasets. It is also used as a predictive modelling tool. It is very popular for solving those problems in which data is contaminated with outliers. It can be applied to problems such as epipolar geometry estimation, model fitting, motions estimation, etc. The default values of all the parameters are used in the implementation.

(h) Simple Linear Regression (SLR)

This is a simple linear regression model that refers to the procedure of minimizing the sum of squared errors. It has one independent variable and one dependent variable, as shown in Equation (12).

(12) $$MIN\;\overset{n}{\underset{i=1}{\sum }}{\left(y_{i}-w_{i}x_{i}\right)}^{2}$$

where *y_i_* is the target variable, *w_i_* is the coefficient, and *x_i_* is the predictor [68]. In this method, we try to minimize the error.

(i) XGBOOST

XGBoost stands for eXtreme Gradient Boosting. It is an execution of a gradient boosted decision tree. XGBoost is popular for its flexibility, speed, and performance compared to other models. This model works best for tabular and structured datasets in classification and regression problems. Parallel processing of XGBoost makes it ten times faster than other tree-based models. It implements regularization to avoid overfitting. The values of some parameters are tuned as colsample_bytree = 0.4, gamma = 0, learning_rate = 0.1, max_depth = 20, min_child_weight = 1.7, n_estimators = 100, reg_alpha = 0.75, reg_lambda = 0.45, subsample = 0.8, seed = 50 and default values for all other parameters are used.

(j) SVM Regressor

SVM is an ML tool used to analyze data, recognize patterns in data, and draw decision boundaries for classification and regression. It constructs hyperplanes in multi-dimensional space that identifies and separates different classes. The number of dimensions is known as a feature vector. SVM can handle multiple categorical and continuous variables. The main purpose of SVM is to minimize the frequency of generalization error by maximizing the perpendicular distance between two sides/edges of different hyperplanes. The generalization potential improves by lowering support vectors as the hyperplane depends on the number of support vectors. We have used the RBF kernel with default parameters.

The execution of these algorithms occurred in python. The default values are used for the parameters whose values are not mentioned in the above descriptions. These are the most popular regression analysis techniques for yield prediction [69] using different parameters.

##### Training and Testing

The input of these regression algorithms was meteorological factors, and the target value was observed tea yield. We had plucking samples of green and black tea for 2016–2019. This data was split into a ratio of 70% and 30%. We have utilized 70% of the data for training the models, and the rest of 30% was used for testing. The mean absolute error (*MAE* (13)), mean squared error (*MSE* (14)), and root mean square error (*RMSE* (15)) were used as objective functions for model performance evaluation and parameterization.

(13) $$MAE=\frac{1}{n}\overset{n}{\underset{j=1}{\sum }}\left|y_{j}-y_{j}\right|$$

(14) $$MSE=\frac{1}{n}\overset{n}{\underset{j=1}{\sum }}{\left(y_{j}-y_{j}\right)}^{2}$$

(15) $$RMSE=\sqrt{\frac{1}{n}\overset{n}{\underset{j=1}{\sum }}{\left(y_{j}-y_{j}\right)}^{2}}$$

In the above equations, $y_{j}$ is the *j*th observed yield and $y_{j}$ is the *j*th predicted yield, and *n* is the total number of samples.

##### 10-Fold Cross-Validation

The 10-fold cross-validation was performed using these meteorological parameters as independent variables and observed yield as the target variable. We have used *MAE*, *MSE*, and *RMSE* as the scoring metrics.

## 4. Results and Discussion

### 4.1. Evaluation of the AquaCrop Model

To evaluate the performance of the AquaCrop model for simulating the *CC*, biomass, and dry yield, different statistical parameters were used: (1) *MAE*, which gives us the indication of the deviation of simulated values from the observed ones [70]. (2) *MSE* error measures the difference between observed and predicted values and calculates the residual sum to eliminate arbitrariness [71]. (3) *RMSE* calculates the discrepancy between simulated and observed values [72].

#### AquaCrop Model Calibration and Validation

In the calibration of AquaCrop, the main parameters are related to *CC*, crop evapotranspiration (ET_a_), biomass, and yield, which need to be calibrated. For the simulation of *CC*, which is a very critical feature of AquaCrop, the main parameters are initial canopy cover (CC_o_), maximum canopy cover (CC_x_), CGC, and cumulative growing degree days (CGDD) in every crop development stage. These parameters were calibrated using data collected during the 2016–2017 growing season. The best values of CC_o_, CCx, and CGC that provide good estimates are 20%, 95%, and 0.7%/day, respectively. Other parameters that affected *CC* development have also been adjusted. The values of CGDD in every crop development stage were determined using base temperature (T_base_) and the upper temperature (T_upper_) equal to 8 °C and 32 °C, respectively, according to NTHRI 2019.

**Figure 4:**
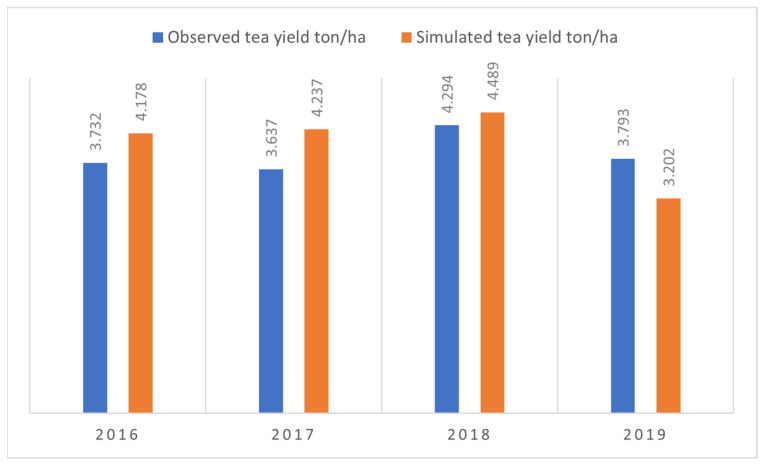
Observed vs. simulated yield for the years 2016–2019.

For the simulation of crop ET_a_, the main parameter was calibrated, affecting soil evaporation and plant transpiration. This parameter is the maximum coefficient for transpiration (*Kc_Tr,x_*). The obtained value of *Kc_Tr,x_* is 0.95, which was adjusted by considering the CCx as 95% for the tea crop. For biomass and final yield simulation, the *HIo* is adjusted equal to *HIo* = 14% and obtained average WP* = 18 g/m^2^, respectively. We have calibrated AquaCrop models using a calibration dataset. Figure 4 shows a comparison of observed and simulated seasonal tea yields in the fields of NTHRI throughout the growing seasons of 2016–2019.

**Figure 5:**
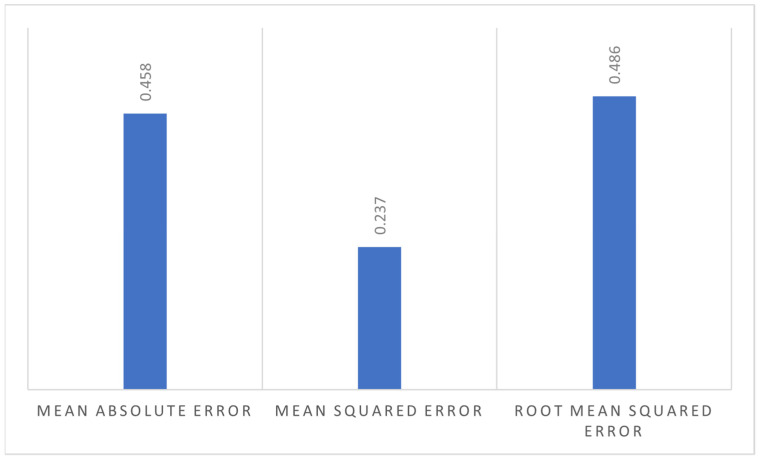
Simulation errors for the years 2016–2019.

Yield values from the AquaCrop model agree well with the observed yield, except in 2019 when the simulated yield is less than the observed yield. This difference can be due to the inconsistent plucking of tea leaves, as the model inputs are highly affected by sampling errors and limitations in data availability [73]. The results can be improved with improved data collection strategies, and many studies discuss data requirements for crop model calibration for different crops [74]. These data show that simulated yields are significantly correlated with the observed yields with *RMSE* of 0.48 t/ha, *MSE* of 0.23 t/ha, and *MAE* of 0.45 t/ha, as shown in Figure 5.

### 4.2. Evaluation of ML Techniques

#### 4.2.1. Train Test Split

**Figure 6:**
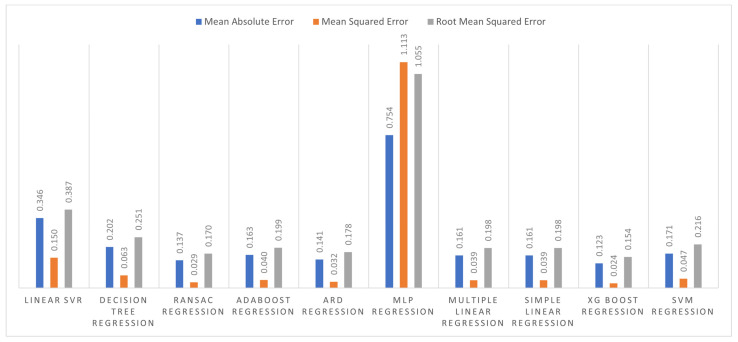
Errors in training and testing of regression algorithms.

MLR models were trained and tested using the above-described dataset. We have trained each ML algorithm with meteorological data as predictors and yield value as a target. These models were trained with 70% of the data set, tested with independent data, and the remaining 30% of the data set. The implementation of these algorithms occurred in python, and three types of error were measured to evaluate their performance, as shown in Figure 6. By analyzing the results of these ML algorithms, we concluded that XGBoost had outperformed all with an *MAE* of 0.123 t/ha, an *MSE* of 0.024 t/ha, and an *RMSE* of 0.154 t/ha on average over the 2016–2019 period. It predicts the yield with a minimum error compared to other algorithms.

**Figure 7:**
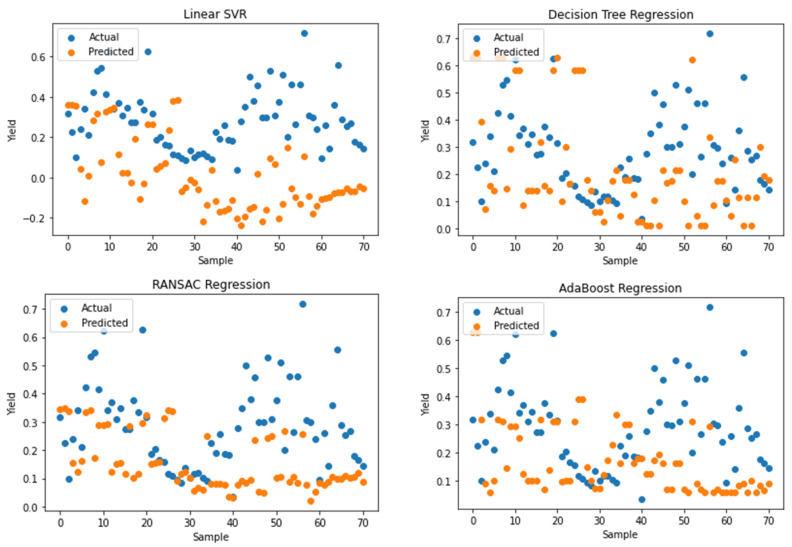

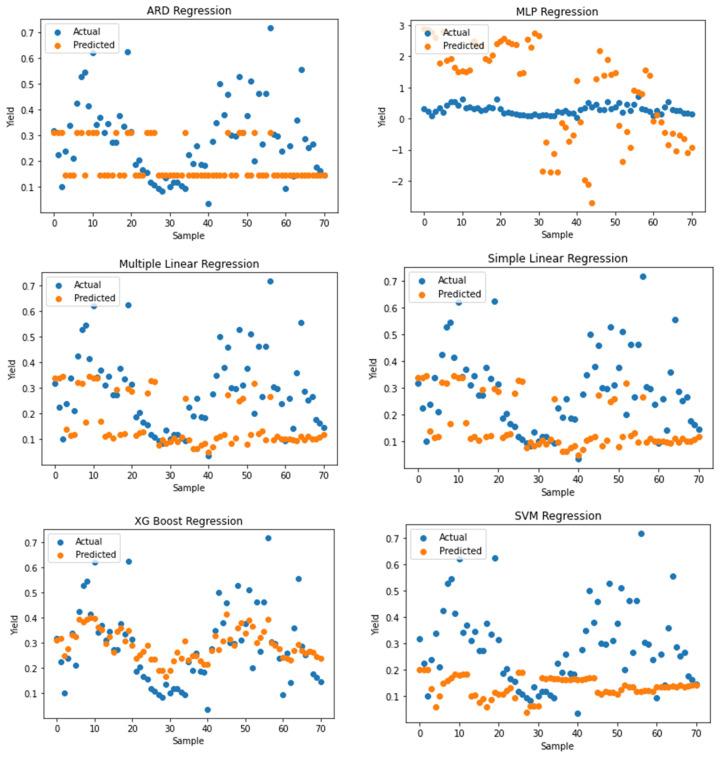
Scatter plots showing actual and predicted yields of all regression algorithms.

The actual yield and predicted yield by these regression algorithms can be visualized by the scatter plots given in Figure 7. We have plotted the sample number on the *x*-axis and the actual and predicted yields on the *y*-axis in different colours.

#### 4.2.2. 10-Fold Cross-Validation

**Figure 8:**
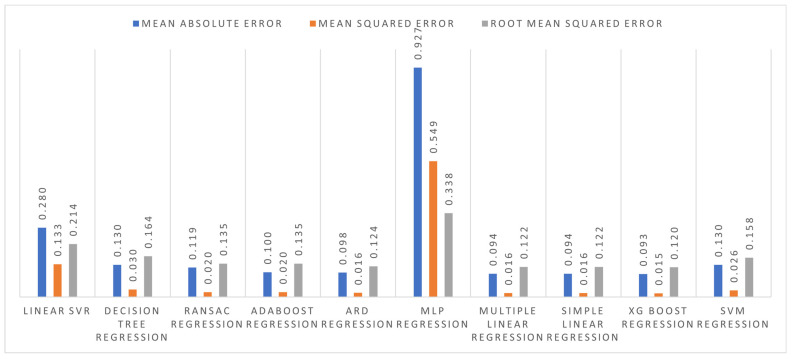
Errors in 10-fold cross-validation of regression algorithms.

The regression models were also implemented using 10-fold cross-validation. Out of these ten regression models, XGBoost performed best with an *MAE* of 0.093 t/ha, an *MSE* of 0.015 t/ha, and an *RMSE* of 0.120 t/ha. The error values for all the models are shown in Figure 8.

Figure 6 and Figure 8 show that 10-fold cross-validation performed well in the train test split method. Table 5 shows the actual yield (kg) and our suggested model’s yield as predicted yield (kg). Out of these 10 regression algorithms, XGBoost shows very promising results.

### 4.3. Comparison of the ML and AquaCrop Model for Predicting Tea Yield

**Figure 9:**
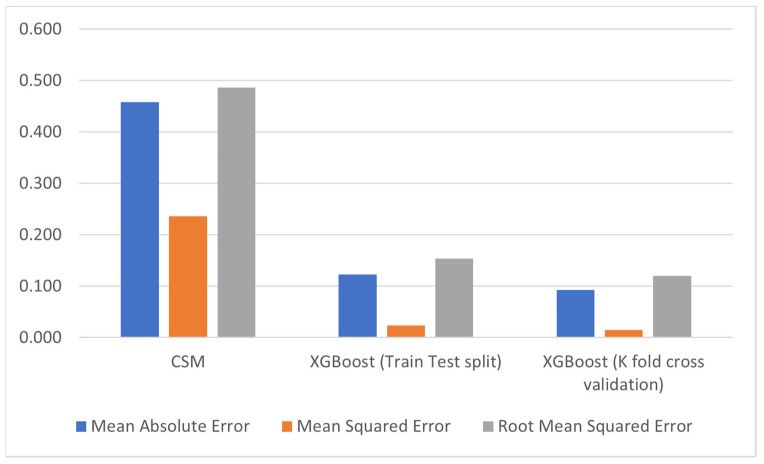
Difference between simulation errors and ML errors.

We have calibrated the AquaCrop simulation model to run simulations for tea yield prediction and applied ML techniques with train test split and 10-fold cross-validation to develop a model for tea yield prediction. Results from both techniques are compared, and the difference between these values is shown in Figure 9. We concluded that the ML models performed better using 10-fold cross-validation than CSM.

To answer our first research question: How to calibrate the selected CSM? We have explained the calibration process of the AquaCrop simulation model. This model was calibrated for tea yield prediction in the following four steps: crop development, crop transpiration, biomass formation, and dry yield. This calibration was conducted using weather, crop, soil, and agro-management data. We have used indicative values of some of the parameters. The results were evaluated using *MAE*, *MSE*, and *RMSE*, which shows a close relation of simulated yield values with observed yield values.

To answer our second research question: How do ML regression algorithms perform for tea yield prediction? We have implemented ten regression algorithms to predict tea yield. These ML techniques require a fewer number of parameters. We used three error measuring techniques: *MAE*, *MSE*, and *RMSE*, to measure the error in observed and predicted yield.

We have also worked to answer our third research question: Which technique from both ML models and simulation models performs better for tea yield prediction? To answer this, we have compared the simulation model’s performance and the ML techniques. Our comparative analysis shows that the ML techniques with 10-fold cross-validation are better than CSMs for yield prediction. Still, these simulation models are useful to run and check different experiments using different climatic, crop, and management practices. The simulation models require a lot of parameters for their calibration [73]. They need climate, soil, crop, and agro-management data for accurate calibration. These parameter values are unavailable in the real world due to recorded data quality.

In comparison to this, ML techniques do not require all these parameters. They can provide good results with a few parameters: temperature, rainfall, humidity, pH, sunshine, etc. If data are available for all parameters, CSMs can be useful for performing and simulating those experiments, which are very expensive and time-consuming.

This study is an important contribution to the use of CSMs for perennial crops and specifically tea crops. It also provides a way to predict tea yield in Pakistan and other areas of the world. It will help increase tea production in Pakistan and other regions by helping to improve management practices using simulation models. These models can provide predictions in different climatic scenarios and environmental conditions.

## 5. Conclusions

This research study acquired data from NTHRI and extracted data from four databases, including weather, crop, soil, and agro-management data. We have selected and calibrated the AquaCrop simulation model for tea yield prediction because it requires fewer parameters than the other simulation models. The AquaCrop model was calibrated using data associated with weather parameters, crop, and soil characteristics to estimate tea crop production. We have trained ML models using this data and observed that the XGBoost regressor outperformed all the other models. We concluded that ML techniques perform better than simulation models by comparing the results of both simulation models and ML techniques. We also concluded that simulation models could be best suited for the estimation of the output of those very expensive and time-consuming experiments if enough input parameters are available. For future studies associated with AquaCrop calibration, it is suggested to use all model variables to perform regression analysis. The results can be further improved by recording more detailed information related to crop and field experiments.

## Figures and Tables

**Table 1:** Summary of related work for different crops.

Paper	Data	Techniques	Crop	Performance
[13]	Weather and crop management	Agroecological Zone simulation model & DM (RF, SVM & GBM)	Sugarcane	*RMSE* ≈ 34 t ha^−1^ *RMSE* ≈ 20.03 t ha^−1^
[55]	Climate and satellite	SVM, RF, ANN	Wheat	R^2^ = 0.75
[51]	Environment and management	A hybrid approach using CNN and RNN	Corn and soybean	*RMSE* ≈ 9%
[43]	Agro-ecological data	AquaCrop	Amaranthus	*RMSE* ≈ 1.96 t ha^−1^
[44]	Soil, weather, crop, and agro-management	AquaCrop	Coffee	R^2^ ≈ 0.71
[58]	Climate, crop, and soil data	Developed a simulation model	Tea	R^2^ > 0.58
[28]	Weather data	Multiple linear models	Tea	Accuracy = 70%

**Table 2:** Weather input data for calibration.

Year	Avg Min Temperature (°C)	Avg Max Temperature (°C)	Avg Humidity (%)	Avg Rainfall (mm)	Solar Radiation (MJ/m^2^)
2016	17.4	32.4	28.5	92.03	19.06
2017	16.7	32.8	33.8	167	19.30
2018	17	32.5	32.64	149.7	19.22
2019	16.8	31.2	55.2	112	19.45

**Table 3:** Soil input data for calibration.

Soil Characteristic	Value
Soil type	Clay loam
Soil depth	2.0 m
Soil water content at soil saturation (θ_SAT_)	50 vol %
Soil water content at field capacity (θ_FC_)	39 vol %
Soil water content at permanent wilting point (θ_PWP_)	23 vol %
Total available soil water (TAW)	160 mm/m
Saturated hydraulic conductivity	500 mm/day
tau (τ) drainage coefficient	0.76

**Table 4:** Crop input data for calibration.

Symbol	Description	Value
	Planting Density (plants/ha.)	12,000
CC_in_	Initial canopy cover after pruning	20%
CC_x_	Maximum canopy cover	95%
CGC	Canopy growth coefficient	
Z_x_	Maximum effective rooting depth (m)	2 m
Z_n_	Minimum effective rooting depth (m)	2 m
WP*	ET water productivity	0.18, 0.18, 0.20, 0.17
*HIo*	Reference harvest index	14%
	Time between pruning	5 years

**Table 5:** An overview of the test data.

Min. Air Temperature	Max. Air Temperature	Average Humidity	Rainfall	Tea Type	Actual Yield (Kg)	Predicted Yield (Kg)
15.9	29.3	54.5	39	Black	275	255.968,9
12.6	34.1	47	39	Black	316	323.608,1
12.6	34.1	47	39.00	Green	186	186.335,9
12.7	33.7	42	39.00	Green	203	184.490,1
12.6	33.9	39	39.00	Green	165	173.613,7
16.6	36	64	35.50	Green	102	99.674,55
20	26	78.5	203.30	Green	120	133.068,1
19.9	33.3	62.5	170.00	Green	143	157.643,5
17.2	33.6	52.5	170.00	Green	164	152.560,5

## Data Availability

The data underlying this article will be shared on reasonable request to the corresponding authors.
